# Is body image misperception associated with sociodemographic factors and life habits? a cross-sectional study 1399 Tunisian school-adolescents

**DOI:** 10.1192/j.eurpsy.2024.972

**Published:** 2024-08-27

**Authors:** F. Mohamed, S. Sellami, D. Amira, J. Fares, G. Rim, M. Jihenne

**Affiliations:** ^1^Department of Psychiatry, Ibn el Jazzar University Hospital, Kairouan, Tunisia, Kairouan; ^2^Department of Psychiatry, Mohamed Taher Maamouri University Hospital, Nabeul, Tunisia, Nabeul; ^3^Department of Epidemiology, Farhat Hached University Hospital, Sousse, Tunisia

## Abstract

**Introduction:**

Understanding adolescents perceptions of their weight status and the factors influencing these perceptions is pivotal for developing targeted interventions and policies to counteract the rising obesity trends.

**Objectives:**

This cross-sectional study aimed to determine the accuracy of weight status perceptions among Tunisian adolescents compared to objective metrics and to identify sociodemographic characteristics and life habits associated with the underestimation of weight status.

**Methods:**

A cross-sectional, school-based study was conducted among a randomized sample of adolescents attending secondary schools in Sousse, Tunisia. A total of 1399 students participated, with anthropometric measurements taken, and a pre-tested Arabic questionnaire administered to gather sociodemographic data and perceived weight status, assessed using the Figure Rating Scale (FRS). The accuracy of perceived weight status was determined by comparing the measured weight status with participants; self-reported perceptions. We evaluated the association between body weight distortion and life habits which included regular physical activity, screen time (time spent on internet per day), number of fruits and vegetables consumed per day, and fast-food consumption.

**Results:**

The study achieved an 86.68% response rate, with over half of the participants being female (60.5%), and the average age being 17 years. The majority of adolescents (41%) perceived themselves as having normal body weight, while 34.5% perceived themselves as underweight, 16.6% as overweight, and 7.9% as obese. However, based on BMI categories, 72.6% had a normal measured weight, 20.4% were overweight, and 6.9% were obese. A substantial proportion of participants (45.6%) underestimated their weight status, with a significant proportion being objectively overweight or obese (26%). Furthermore, we found a significant association between the perception of weight accuracy with four correlates: gender, mother educational level, regular physical activity, and the number of fruits and vegetables consumed per day.

**Conclusions:**

The findings revealed a disparity between perceived and actual weight status among Tunisian adolescents, with a significant underestimation of weight status, particularly among those who are overweight or obese. The results highlighted the crucial need for interventions that address weight perception inaccuracies and promote healthy weight awareness and management among adolescents in Tunisia. The study underscored the importance of further research to understand the development and progression of body weight underestimation throughout adolescence and the roles of lifestyle behaviors in shaping weight perceptions.

**Disclosure of Interest:**

None Declared

